# The Noninvasive Retro-Mode Imaging Modality of Confocal Scanning Laser Ophthalmoscopy in Polypoidal Choroidal Vasculopathy: A Preliminary Application

**DOI:** 10.1371/journal.pone.0075711

**Published:** 2013-09-18

**Authors:** Renpan Zeng, Xiongze Zhang, Yu Su, Meng Li, Kunfang Wu, Feng Wen

**Affiliations:** State Key Laboratory of Ophthalmology, Zhongshan Ophthalmic Center, Sun Yat-sen University, Guangzhou, China; Massachusetts Eye & Ear Infirmary, Harvard Medical School, United States of America

## Abstract

**Purpose:**

To evaluate the validity of the novel and noninvasive retro-mode imaging modality of confocal scanning laser ophthalmoscopy (cSLO) for detecting the morphological features of polypoidal choroidal vasculopathy (PCV).

**Design:**

Prospective, observational, consecutive case series.

**Methods:**

Twenty-six patients (29 eyes) with PCV were enrolled in this study. All patients underwent comprehensive ophthalmologic examinations and imaging studies, including retro-mode imaging, fundus autofluorescence (FAF), fundus photography, fundus fluorescein angiography (FFA), indocyanine green angiography (ICGA) and spectral-domain optical coherence tomography (SD-OCT). We investigated the retro-mode images and compared the results with those of SD-OCT, FFA and ICGA.

**Results:**

In the 29 PCV eyes, the retro-mode images clearly revealed polypoidal lesions in 27 (93.1%) eyes as well as branching vascular networks in 16 (55.2%) eyes. Others findings, including pigment epithelial detachment (PED) in 20 (69.0%) eyes, neuroretinal detachment (NRD) in 3 (10.3%) eyes, cystoid macular edema (CME) in 3 (10.3%) eyes, drusen in 4 (13.8%) eyes and minute granular changes of the retinal pigment epithelium (RPE) in 12 (41.3%) eyes, were also clearly visualized. When we compared the results with those of SD-OCT, FFA and ICGA, there was no significant difference between ICGA and retro-mode imaging for finding polypoidal lesions and (or) branching choroidal vascular networks (P>0.05). However, the rate of PED detection was significantly better with retro-mode imaging than with the ICGA (P<0.05). The differences were not statistically significant between SD-OCT and retro-mode imaging for detecting PED, NRD, CME, drusen and minute granular RPE changes (P>0.05). The differences were not statistically significant between FFA and retro-mode imaging for detecting PED, NRD, CME (P>0.05).

**Conclusions:**

The novel and noninvasive retro-mode imaging by cSLO is able to clearly visualize the morphological features of PCV.

## Introduction

Polypoidal choroidal vasculopathy (PCV) is a specific form of choroidal vasculopathy associated with multiple, recurrent serosanguineous detachments of the retinal pigment epithelium (RPE) and neurosensory retina [1,2]. The most effective way to establish a definitive diagnosis of PCV is through indocyanine green angiography (ICGA); the diagnosis of PVC is mainly based on the presence of branching choroidal vascular networks with polyp-like terminal aneurysmal dilations or scattered polypoidal dilations without identifiable continuous branching vascular networks in ICGA [3,4]. However, conventional ICGA is an invasive imaging modality. It requires the injection of intravenous dye and a long capture time. Spectral-domain optical coherence tomography (SD-OCT) is currently one of the best available noninvasive imaging tools for the management of PCV [5,6]. Previous studies based on SD-OCT have demonstrated morphologic abnormalities surrounding the polypoidal lesions [7,8] in most PCV cases, such as pigment epithelial detachment (PED) and protruding or irregular RPE. Recent studies using high-resolution OCT have clearly demonstrated that polypoidal structures and branching networks of vessels existed beneath the RPE [9,10]. However, SD-OCT has some limitations regarding the detection of the entire polypoidal lesion and branching choroidal vascular network underlying the RPE and all of the PCV-related abnormalities of the RPE in the posterior fundus pole. Essentially, SD-OCT provides cross-sectional images of a limited area, rather than comprehensive topographic fundus images. Fundus autofluorescence (FAF), another noninvasive imaging modality, provides topographic information about the metabolic activity of the RPE [11]. However, FAF does not provide detailed information about the anatomic abnormalities of the choroidal vascular and RPE. Therefore, specific protocols for scanning the entire macula and showing the whole PCV lesions are still required.

Infrared imaging has been used for the visualization of deeper retinal structures because it can penetrate the deeper layers [12]. Recently, based on the principles of retro-illumination, retro-mode imaging with infrared lasers (Nidek F-10, Gamagori, Japan) confocal scanning laser ophthalmoscopy (cSLO) was used to investigate several fundus pathologies, including cystoid macular edema [13], myopic retinoschisis [14], drusen [15], subthreshold laser scars [16], and RPE alterations in central serous chorioretinopathy [17]. These studies implied that retro-mode imaging could be useful in the study of deep retinal pathologies, RPE changes and even choroidal lesions. However, to the best of our knowledge, there are no reports describing retro-mode imaging of various choroidal morphologic characteristics in patients with PCV. Accordingly, the aim of this study was to evaluate noninvasive retro-mode imaging by cSLO as a novel imaging modality for detecting choroidal morphologic features of PCV. Moreover, we also sought to compare the findings obtained using this approach with those obtained by other imaging modalities (FFA, ICGA and SD-OCT).

## Methods

### Ethics Statement

The study protocol adhered to the tenets of the Declaration of Helsinki. The institutional review board at the Zhongshan Ophthalmic Center of Sun Yat-sen University approved this study and data accumulation (lot number: 2012KYNL015). Written informed consent for the research was obtained from the patients after explanation of the purpose and process of the study.

### Inclusion and Exclusion Criteria

We performed a prospective study to enroll the PCV patients who attended the macular service of the Zhongshan Ophthalmic Center (Guangzhou, China) for a clinical evaluation from January 2012 to June 2012. All patients underwent comprehensive ophthalmologic examinations and imaging studies, including retro-mode imaging, FAF, fundus photography, fundus fluorescein angiography (FFA), ICGA and SD-OCT. All imaging studies were performed on subjects with dilated pupils. We used ICGA to make a definitive diagnosis of PCV in each patient; this diagnosis was based on the presence of polypoidal lesions with or without branching vascular networks in ICGA that are characteristics of the disease [3,18]. Patients with other neovascularised maculopathies, such as neovascular age-related macular degeneration, retinal angiomatous proliferation, angioid streaks, pathological myopia, presumed ocular histoplasmosis, and other retinal or choroidal diseases that could account for CNV were excluded from the study. Additionally, patients with dense cataracts were excluded because of the difﬁculty in obtaining clear images.

### Image Acquisition

Prior to beginning the FFA and ICGA, retro-mode imaging and FAF of the posterior poles were performed using the F-10 (Nidek Co, Gamagori, Japan) with an image field of 40 degrees, an optical resolution of 16 to 20 µm and an image size of up to 1024x720 pixels. For the retro-mode imaging, infrared laser (790 nm) light was used to scan the fundus because of its ability to penetrate deeper layers. Two different retro-mode images per eye were consecutively obtained via a right-deviated aperture and a left-deviated aperture. In the FAF mode, the device used blue light (490 nm) to capture 20 FAF images per eye, and the images were synthesized into one image using NAVIS-Lite software. The fovea was centrally located in all FAF images.

Fundus photography, FFA and ICGA were performed in both eyes of each patient using a Zeiss FF450 plus fundus camera (Carl Zeiss, Inc., Jena, Germany) [18]. Informed consent was obtained from all of the patients before they underwent the FFA and ICGA examinations. For the angiography procedures, sodium fluorescein and indocyanine green (ICG), were injected into the antecubital vein (for FFA and ICGA, respectively), and the timing was begun. The angiograms were obtained over 15 minutes for FFA and over 30 minutes for ICGA. The cross-sectional OCT images were obtained by SD-OCT (Spectralis, Heidelberg Engineering, Heidelberg, Germany). We used both vertical and horizontal cross-sectional OCT scans; the axial and transverse resolutions are 7 and 20 µm respectively, and an automated tracking function was employed during scanning. All images were stored as high-quality tif ﬁles.

### Identification of Clinical Features Associated with PCV

All images were separately reviewed by at least two of the authors to identify and record various morphologic alterations associated with PCV to interpret each lesion for the study. The discrepancies were referred to a fundus specialist (FW) for final determination. Evidence of polypoidal lesions in ICGA and retro-mode imaging was defined by the presence of polyp-like dilations [2], while branching vascular networks was defined by the presence of inner choroidal vascular abnormalities that showed focal dilation, constriction, and tortuosity of choroidal vessels [19] in ICGA and retro-mode imaging. Evidence of PED, NRD, CME, drusen and minute granular RPE changes in FFA, ICGA and SD-OCT were defined by the criteria used in previous studies [15,20,21,22]. In retro-mode imaging, PED was defined as a well-demarcated prominence with a dark shadow [17], NRD was defined as a translucent ﬂat elevated area [20], CME was defined like a honeycomb feature with large cells in the fovea [21], drusen was defined as well-demarcated scattered convex deposits [15], minute granular RPE changes were defined as well-demarcated, ragged surface area [22].

Statistical analysis of the data was performed using the SPSS software package (version 16.0, SPSS Inc., Chicago, Illinois, USA). Chi-square testing was performed for categorical analysis. The Fisher exact test was performed if the expected cell count was less than 5. A P value of less than 0.05 was considered to be statistically significant.

## Results

### Demographic data and baseline characteristics

Twenty-six patients (29 eyes) with PCV were enrolled in this study. There were 16 men (18 eyes) and 10 women (11 eyes) aged from 47 to 78 years (average: 63.0 ± 7.9 years).

### Retro-mode imaging findings

**Figure 1:**
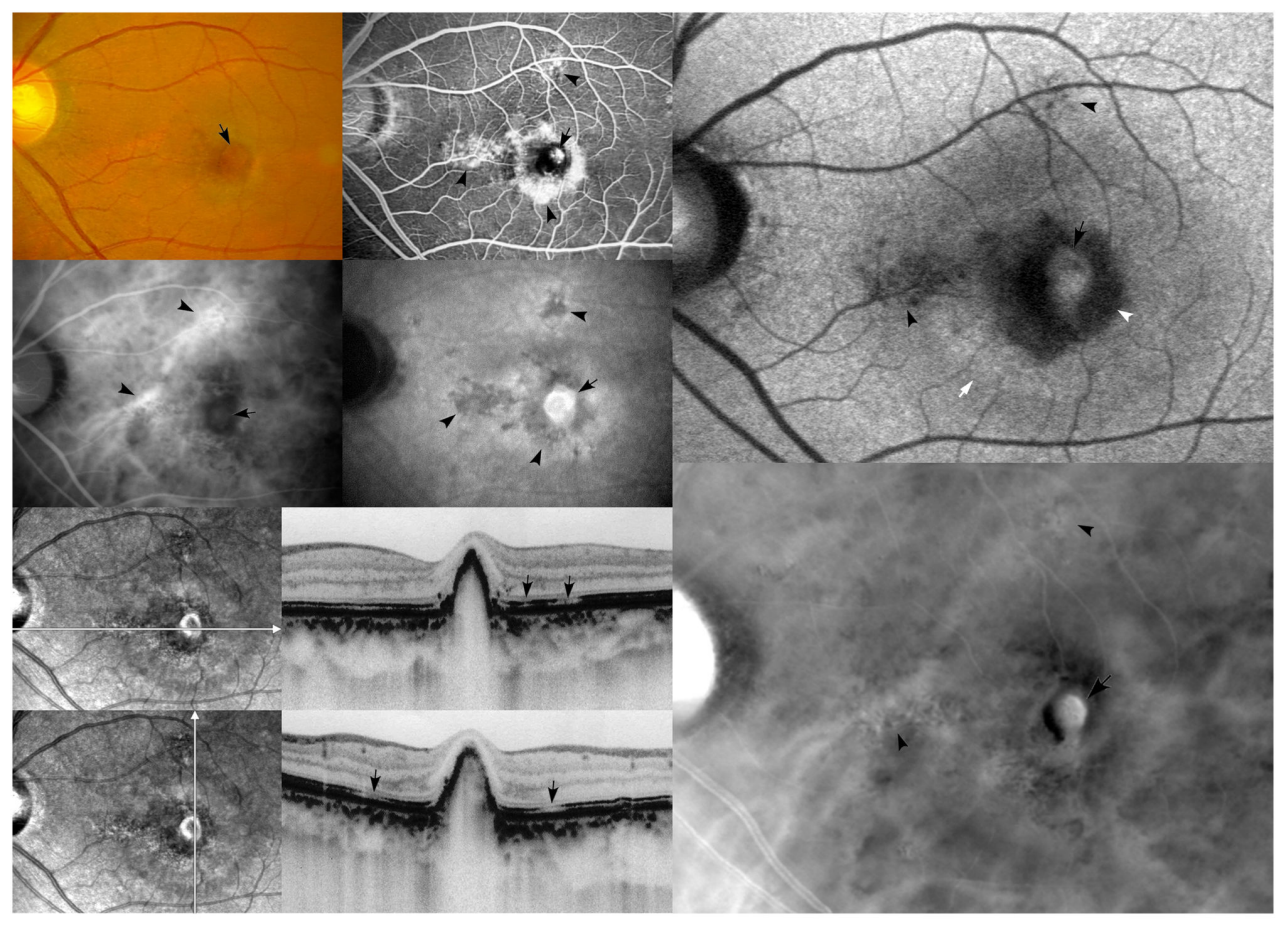
A representative case of polypoidal choroidal vasculopathy with a single polypoidal lesion. (Top left) A fundus photograph shows a reddish-orange lesion (arrow) surrounded by subretinal hemorrhage and retinal pigment epithelium depigmentation in the macular area (Top middle). FFA shows saccular hyperfluorescence (arrow) and irregular transmitted fluorescence (arrowheads) in the macular area (Middle left). Early phase of ICGA shows a polypoidal lesion, saccular slight hyperfluorescence (arrow) and dilated choroidal blood vessels (arrowheads) in the macular area (Middle middle). The late phase of ICGA shows the “washout” of a polypoidal lesion (arrow) and hypofluorescence region (arrowheads) corresponding with transmitted fluorescence in the FFA (Bottom left). Both horizontal and vertical cross-sectional OCT scans show a localized, steep, protruded polypoidal lesion and the disruption of the outer photoreceptor layer (arrows) (Top right). FAF shows granular hypoautofluorescence (black arrowheads) and confluent hypoautofluorescence (white arrowhead), which correspond to the transmitted fluorescence in FFA and the hypofluorescence region in ICGA. Moreover, that FAF shows an oval-shaped hyperautofluorescence (black arrow) at the site of the polypoidal lesion and a hyperautoﬂuorescent ring (white arrow) surrounding the conﬂuent hypoautoﬂuorescence (white arrowhead) (Bottom right). Retro-mode imaging shows a well-defined polypoidal lesion (arrow) and an irregular, well-defined, ragged surface area (arrowheads), which correspond well with the ICGA.

**Figure 2:**
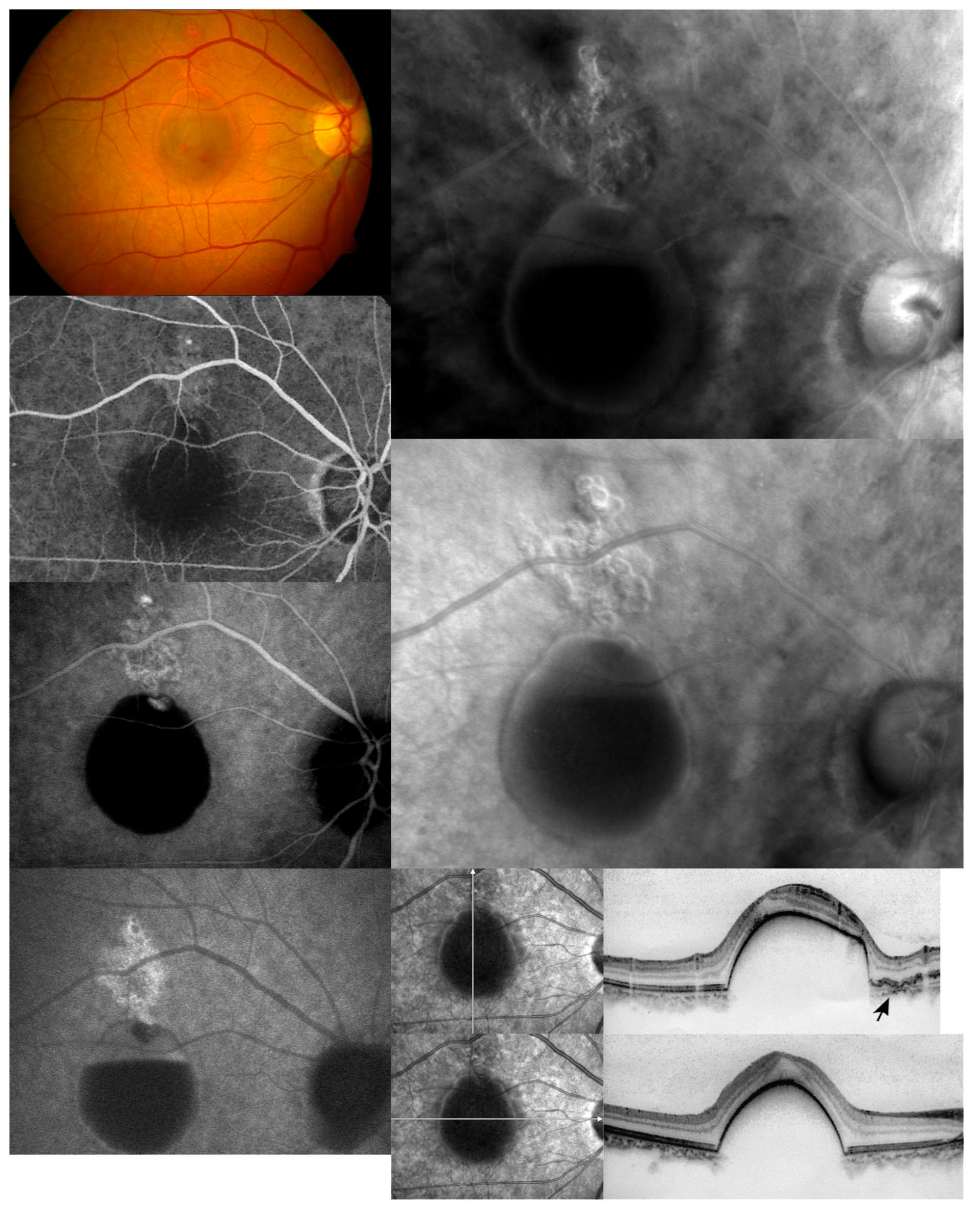
A representative case of polypoidal choroidal vasculopathy with cluster polypoidal lesions in retro-mode imaging. (Left column) From top to bottom are the fundus photograph, FFA and the early and late phase of ICGA. The fundus photograph shows reddish-orange lesions with a serosanguineous retinal pigment epithelium detachment in the macular area. The FFA shows occult CNV and a subretinal hemorrhage. The early phase of the ICGA shows polypoidal lesions with branching choroidal vascular networks and oval PED. The late phase of the ICGA shows the “washout” of the polypoidal lesions and leakage from branching choroidal vascular networks (arrow) and serosanguineous retinal pigment epithelium detachment (Right column, top). Retro-mode imaging using the right-deviated aperture shows well-defined cluster polypoidal lesions and serosanguineous retinal pigment epithelium detachment with a convex appearance (Right column, middle). Retro-mode imaging using the left-deviated aperture shows a well-defined cluster polypoidal lesions and serosanguineous retinal pigment epithelium detachment with a concave appearance (Right column, bottom). Both vertical and horizontal cross-sectional OCT scans show a large, steep, hemorrhagic PED. The vertical cross-sectional OCT scan shows an irregular RPE (arrow) above the hemorrhagic PED.

**Figure 3:**
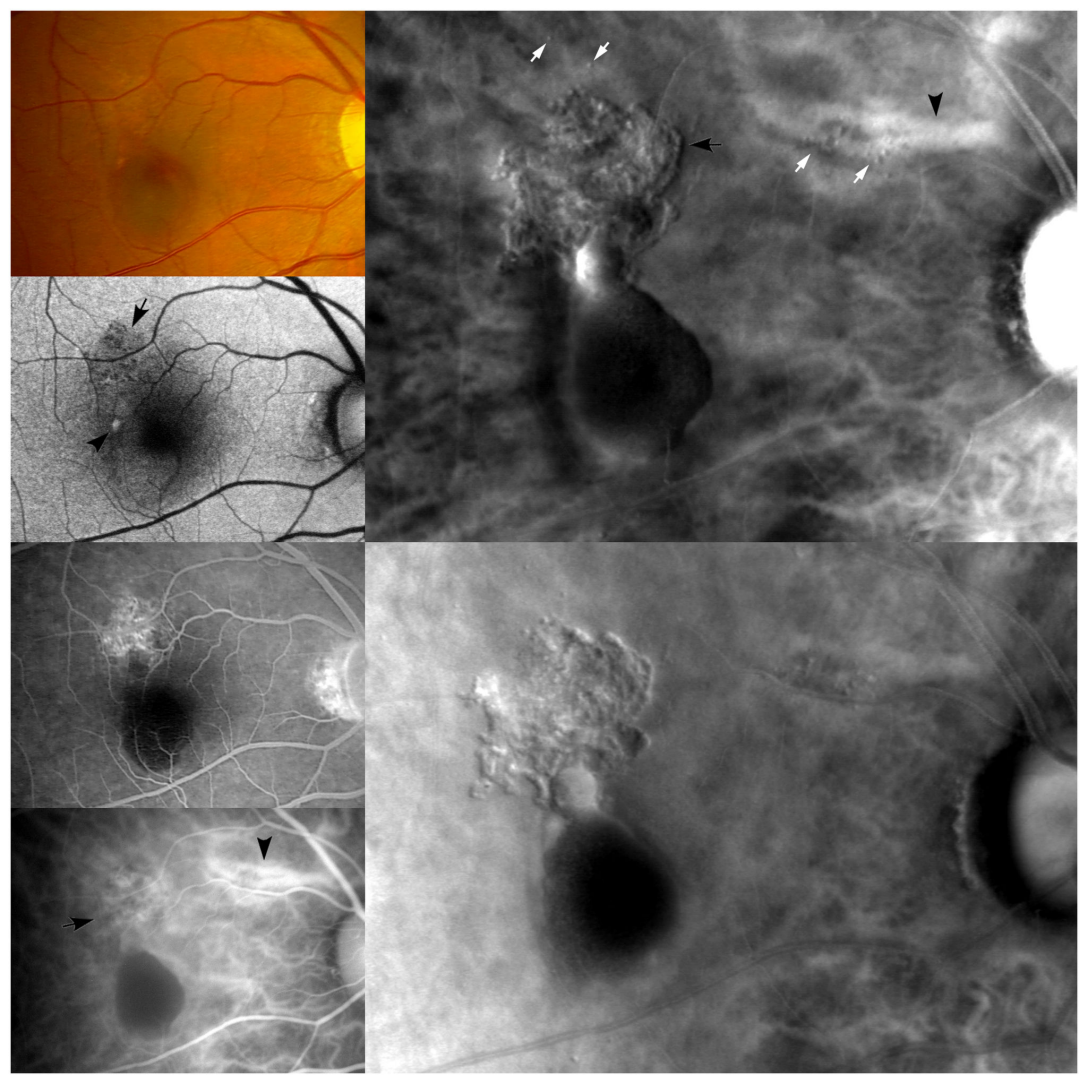
A representative case with polypoidal lesions and branching choroidal vascular networks in retro-mode imaging. (Left column) From top to bottom, a fundus photograph, FAF, FFA and ICGA. The fundus photograph shows retinal pigment epithelium depigmentation and subretinal hemorrhage in the macular area. The FAF shows granular hypoautofluorescence (arrow) corresponding with the retinal pigment epithelium depigmentation in the fundus photograph. Moreover, the FAF shows a hyperautofluorescence (arrowhead) in the macular area. The FFA shows an occult CNV and subretinal hemorrhage. The ICGA shows polypoidal lesions with branching choroidal vascular networks (arrow) and oval PED in the macular area and the dilated choroidal blood vessels (arrowhead) at the super-temporal area of the optic disc (Right column, top). Retro-mode imaging using the right-deviated aperture shows well-defined polypoidal lesions with branching choroidal vascular networks (black arrow), hemorrhagic retinal pigment epithelium detachment, drusen (white arrows) and dilated choroidal blood vessels (black arrowhead) with a convex appearance (Right column, bottom). Retro-mode imaging using the left-deviated aperture shows well-defined polypoidal lesions with branching choroidal vascular networks, hemorrhagic retinal pigment epithelium detachment, drusen and dilated choroidal blood vessels with a concave appearance.

**Figure 4:**
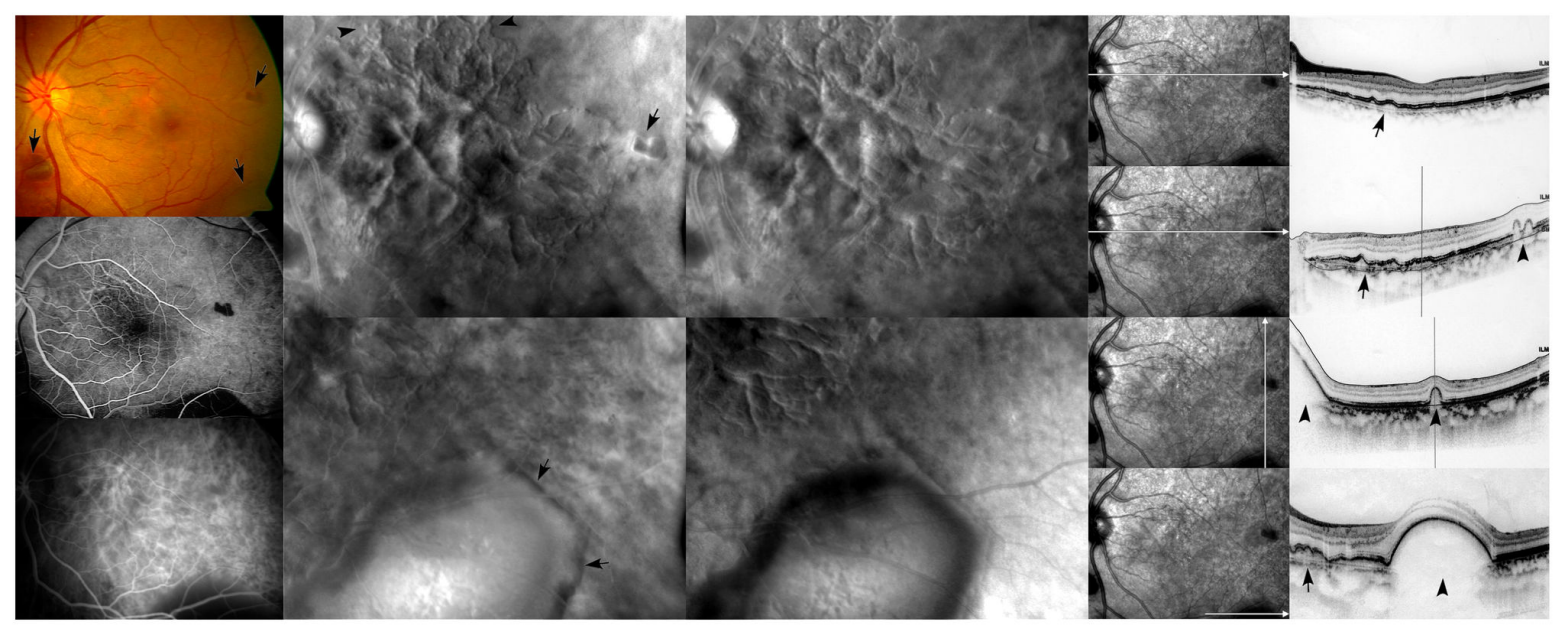
A representative case with polypoidal lesions and branching choroidal vascular networks in retro-mode images. (Left column) From top to bottom, a fundus photograph, FFA and ICGA. The fundus photograph shows several subretinal hemorrhages (arrows). The FFA shows branching choroidal vascular networks in the macular area and a subretinal hemorrhage corresponding with the fundus photograph. The ICGA shows polypoidal lesions with branching choroidal vascular networks in the macular area (Second column). Retro-mode imaging using the right-deviated aperture shows well-defined polypoidal lesions with branching choroidal vascular networks (arrowheads) and hemorrhagic retinal pigment epithelium detachment (arrows) with a convex appearance (Third column). Retro-mode imaging using the left-deviated aperture shows well-defined polypoidal lesions with branching choroidal vascular networks and hemorrhagic retinal pigment epithelium detachment with a concave appearance (Right column). SD-OCT scans show hemorrhagic PED (arrowheads) and irregular RPE (arrows) corresponding to abnormal branching choroidal vascular networks.

In the 29 PCV eyes, the retro-mode images clearly revealed polypoidal lesions in 27 (93.1%) eyes and branching vascular networks in 16 (55.2%) eyes. A single polypoidal lesion without branching vascular networks (Figure 1, Bottom right) was detected in 3 eyes, cluster polypoidal lesions without branching vascular networks (Figure 2, Right column, top and middle) were found in 8 eyes, polypoidal lesions with branching vascular networks (Figure 3, Right column; Figure 4, Second column and Third column) were found in 16 eyes. Other findings, including pigment epithelial detachment (PED) (Figure 2, Figure 3, Figure 4) in 20 (69.0%) eyes, neuroretinal detachment (NRD) in 3 (10.3%) eyes, cystoid macular edema (CME) in 3 (10.3%) eyes, drusen (Figure 3) in 4 (13.8%) eyes and minute granular RPE changes (Figure 1) in 12 (41.3%) eyes, were also clearly visualized.

### Description of morphologic features of PCV using retro-mode imaging

Using the right-deviated aperture, the polypoidal lesions, branching vascular networks, PED, drusen, NRD, and CME revealed by retro-mode imaging had a well-defined, convex appearance with lighter zone on the right side and a darker zone on the left side. In contrast, when the left-deviated aperture was used, these features had a well-defined, concave appearance with a lighter zone on the left side and a darker zone on the right side. The minute granular RPE changes revealed by retro-mode imaging appeared as a well-defined, ragged surface area.

### FAF findings

FAF showed granular or confluent hypoautofluorescence (Figure 3) at the sites of polypoidal lesions and branching choroidal vascular networks in 24 (82.8%) eyes, and oval hyperautofluorescence (Figure 1, Figure 3) was observed at the site of polypoidal lesion in 2 (6.9%) eyes. In addition, granular or irregular or confluent hypoautofluorescence was also observed outside the site of the polypoidal lesions and branching choroidal vascular networks (Figure 1) in 7 (24.1%) eyes, which corresponded with the minute granular RPE changes. A hyperautoﬂuorescent ring (Figure 1) surrounding conﬂuent hypoautoﬂuorescence was observed in 4 (13.8%) eyes.

The comparison of the findings obtained using retro-mode imaging with those obtained using other imaging modalities (FFA, ICGA and SD-OCT) in the 29 PCV eyes is shown in Table 1. There were no significant difference between ICGA and retro-mode imaging in the detection of polypoidal lesions and (or) branching choroidal vascular networks (P>0.05). However, the rate of the PED detection was significantly better with retro-mode imaging than with the ICGA (P<0.05). The differences were not statistically significant between SD-OCT and retro-mode imaging for detecting PED, NRD, CME, drusen and minute granular RPE changes (P>0.05). The differences were not statistically significant between FFA and retro-mode imaging for detecting PED, NRD, CME (P>0.05).

**Table 1:** Comparison of the findings obtained by retro-mode imaging with those obtained by other imaging modalities (fundus fluorescein angiography, indocyanine green angiography and spectral-domain optical coherence tomography) in the 29 PCV eyes; n (%).

	FFA	ICGA	SD-OCT	Retro-mode
Polypoidal lesions	-—	29 (100.0)^a^	-—	27 (93.1)
Branching vascular networks	-—	17 (58.6)^a^	-—	16 (55.2)
PED	16 (55.2)^a^	12 (41.3)^b^	22 (75.9)^a^	20 (69.0)
NRD	2 (6.9)^a^	1 (3.4)^a^	5 (17.2)^a^	3 (10.3)
CME	3 (10.3)^a^	0 (0.0)^a^	3 (10.3)^a^	3 (10.3)
Drusen	-—	-—	4 (13.8)^a^	4 (13.8)
Minute granular RPE changes	-—	-—	10 (34.5)^a^	12 (41.3)

Compared with Retro-mode: ^a^P>0.05; ^b^P<0.05

FFA = fundus fluorescein angiography; ICGA = indocyanine green angiography; SD-OCT = Spectral-domain optical coherence tomography; PED = pigment epithelial detachment; NRD = neuroretinal detachment; CME = cystoid macular edema; RPE = retinal pigment epithelium

The detailed clinical profiles and findings of retro-mode imaging, FAF, FFA, ICGA and SD-OCT are shown in the Table S1.

## Discussion

Retro-mode imaging has recently been shown to offer the best method for outlining the distinctive clinical features of several chorioretinal disorders. The detailed principles of retro-mode imaging are well described in the literature [14,15,16,17,21,23,24,25]. In brief, retro-mode imaging is performed by a cSLO working at an infrared wavelength to produce a pseudo-3D image. It is equipped with a retro-mode aperture consisting of a central stop associated with a laterally oriented oval-shaped opening, which prevents direct light reflection from crossing and allows the scattered light from only one direction to pass through the lateral aperture. The detector acquires images from the surface to the deep retinal layers through the lateral aperture and produces a clear image appearing as a shadow of the silhouetted retinochoroidal details. The shadows of the lesions appear differently according to the laterality of the annular aperture. Both right-deviated and left-deviated apertures were used in this study. This imaging technique offers a new means of detecting abnormalities in the deeper retinal and choroid tissue.

In previous studies, retro-mode imaging was used to characterize the CME associated with different diseases, including diabetic retinopathy [21,24,26], retinal vein occlusion [21,26], uveitis [21,26], age-related macular degeneration (AMD) [21,25,26], PCV [27] and retinitis pigmentosa [26]. Shin et al. reported comprehensive topographic descriptions of RPE alterations in central serous chorioretinopathy using the retro-mode imaging [17]. Tanaka et al. have described a specific “fingerprint” pattern related to macular retinoschisis in myopic eyes, which consists of radiating retinal striae centered on the fovea and many light dots and lines that run parallel to the striae or form a whorled pattern surrounding the radiating striae [14]. Meanwhile, in eyes with X-linked retinoschisis, retro-mode imaging clearly delineated the stellate spoke-like foveal schisis [28]. Jennifer et al. reported that retro-mode imaging featured the drusen in eyes affected by dry age-related macular degeneration, which were consistent with the appearance of drusen on OCT imaging, and retro-mode imaging detected signiﬁcantly more drusen than conventional color fundus photography [15]. In another study, retro-mode imaging was used to detect laser spots created by subthreshold diode laser micropulse photocoagulation, which presented as dark spots. This method is useful to conﬁrm the invisible spots created by subthreshold diode laser micropulse photocoagulation [16]. Finally, retro-mode imaging was also a useful tool for characterizing retinal dystrophies [23,29]. It provided additional information on the clinical setting and the monitoring of these diseases.

In the current study, our results showed that retro-mode imaging could clearly reveal the features of polypoidal lesions, branching vascular networks, PED, NRD, CME, drusen and minute granular RPE changes in PCV eyes. These findings indicate that retro-mode imaging is a useful means for detecting the choroidal morphologic features of PCV. Moreover, retro-mode imaging detected subtle PED, CME and minute lesions of the RPE, which were invisible on ICGA. However, in some cases with severe CME in the macular area, the polypoidal lesions and branching vascular networks could not be detected clearly by retro-mode imaging because severe CME interfered with the visualization by blocking the effects.

In eyes with PCV, ICGA is conventionally used to detect polypoidal lesions and branching vascular networks, SD-OCT is used to detect PED, NRD, CME, drusen and RPE changes, which provides cross-sectional images, not comprehensive topographic fundus images. In the current study, our results demonstrated that there were no significant differences between ICGA and retro-mode imaging for detecting polypoidal lesions and (or) branching choroidal vascular networks, and the differences between SD-OCT and retro-mode imaging for detecting PED, NRD, CME, drusen and minute granular RPE changes (minute RPE protrusion) were also not statistically significant. Moreover, retro-mode imaging represents a new, noninvasive means to scan the entire macula and shows the comprehensive PCV lesions, as opposed to cross-sectional images. However, retro-mode imaging alone was unable to differentiate flat PED and NRD from RPE irregularities.

With recent improvements of OCT, more detailed PCV lesions can be observed. Recent studies have demonstrated that between the RPE line and Bruch’s membrane, various PCV lesions, including vascular networks, polypoidal lesions, and sub-RPE hemorrhage were clearly identified in cross-sectional images by both eye-tracked SD-OCT modality [10] and OCT with an enhanced depth imaging modality [30]. Moreover, SD-OCT with the en-face scan modality can produce a 3-dimensional imaging of the RPE contour [31]. However, it is still difficult to distinguish the vascular networks, polypoidal lesions and hemorrhage elements that beneath the RPE by 3-dimensional SD-OCT. In view of the shortcomings of SD-OCT, we suggested that SD-OCT and retro-mode imaging may thus be considered complementary techniques, together providing a fast and noninvasive examination of the fundus.

FAF is considered to be related to the amount of lipofuscin within the RPE cells and provides information about RPE metabolism and function [32]. In this study, FAF showed granular hypoautofluorescence at the site of the polypoidal lesions and branching choroidal vascular networks in most PCV eyes (82.8%), as well as irregular hypoautofluorescence outside the site of the polypoidal lesions and branching choroidal vascular networks in some PCV eyes (13.8%), which is consistent with a previous study [33]. Moreover, in our study, FAF showed oval hyperautofluorescence at the site of single polypoidal lesions in a few PCV eyes (6.9%), which reflected the corresponding impairment of RPE metabolism.

Our results revealed that retro-mode imaging was also able to detect other abnormalities, such as PED, NRD, CME, drusen and minute granular RPE changes in PCV eyes. All these lesions were in a pseudo-3 dimensional pattern at the posterior pole and appeared as elevated areas with different shapes and sizes. Moreover, retro-mode imaging provided more precise imaging of the elevated lesions than biomicroscopic examination, precisely detecting even small alterations that may be missed in a simple ophthalmoscopic examination.

In addition, we found that retro-mode imaging was more effective than FFA and ICGA for characterizing deep retinal RPE and choroid abnormalities in PCV. Generally, FFA depicts PCV simply as occult CNV, but it does not reveal detailed characteristics. Meanwhile, ICGA has the advantage of evaluating choroidal abnormalities, but it provides limited information about RPE abnormalities. On this aspect, retro-mode imaging can provide comprehensive information about PCV.

Retro-mode imaging has several advantages in the detection of choroid and RPE alterations in PCV. It is a noninvasive, rapid, and simple imaging technique. The imaging process is similar to that of SD-OCT and fundus photography. When compared with FFA and ICGA, retro-mode imaging does not require a long capture time and the injection of intravenous dye. Another advantage is the infrared laser used in retro-mode imaging. The examination can be performed under nonmydriatic situations or even in cases with significant lens opacity because of the properties of a long-wavelength laser. Moreover, patients may feel comfortable during the examination because the infrared light is less irritating than the blue light used in FFA. With respect to the use of retro-mode imaging for detecting various PCV lesions, several issues should be considered. First, retro-mode imaging is currently available only with the use of the Nidek F-10. The investigation of retro-mode imaging is in its earliest stages; there are some shortcomings related to the interpretation of these findings. Second, this technique cannot replace SD-OCT, FFA, and ICGA in evaluating PCV. SD-OCT should still be considered as the gold standard for diagnosing anatomic lesions in different fundus layers. In contrast to FFA and ICGA, retro-mode imaging could not be used to describe the disease activity of PCV. It is difficult to make a treatment decision using retro-mode imaging. Therefore, retro-mode imaging may be helpful as an imaging technique to complement FFA, ICGA, and SD-OCT. Third, a small sample size and no follow-up data are major limitations of this study. Future prospective studies with large sample sizes should be considered to determine the effectiveness of retro-mode imaging in the diagnosis and follow-up of PCV patients. We propose retro-mode imaging as another noninvasive diagnostic tool for detecting various PCV lesions. This technique is capable of providing comprehensive topographic information related to the choroid and RPE alterations.

## Supporting Information

Table S1 **Clinical profiles and characteristics of fundus autofluorescence, fundus fluorescein angiography, indocyanine green angiography, spectral-domain optical coherence tomography and retro-mode imaging in the 29 PCV eyes.**
(DOC)
