# Supplementary material for: The Noninvasive Retro-Mode Imaging Modality of Confocal Scanning Laser Ophthalmoscopy in Polypoidal Choroidal Vasculopathy: A Preliminary Application
Source: PLoS One. 2013 Sep 18;8(9):e75711. doi: 10.1371/journal.pone.0075711 (PMC3776759; doi:10.1371/journal.pone.0075711)
Supplement: Table S1 — Clinical profiles and characteristics of fundus autofluorescence, fundus fluorescein angiography, indocyanine green angiography, spectral-domain optical coherence tomography and retro-mode imaging in the 29 PCV eyes. (DOC) [file pone.0075711.s001.doc]

**Table S1:** Clinical profiles and characteristics of fundus autofluorescence, fundus fluorescein angiography, indocyanine green angiography, spectral-domain optical coherence tomography and retro-mode imaging in the 29 PCV eyes.

| Case No. | Sex | Age(year) | FAF | | | | |
| --- | --- | --- | --- | --- | --- | --- | --- |
|  |  |  | Granular  hypoautofluorescence | Oval  hyperautofluorescence | confluent  hypoautofluorescence | hyperautoﬂuorescent  ring | Irregular  hypoautofluorescence |
| 1 | M | 63 | ＋ | － | ＋ | ＋ | ＋ |
| 2 | M | 61 | ＋ | ＋ | － | － | － |
| 3 | F | 57 | ＋ | － | － | － | － |
| 4 | M | 78 | ＋ | － | ＋ | ＋ | ＋ |
| 5 | F | 71 | ＋ | － | － | － | － |
| 6 | F | 73 | ＋ | － | － | － | － |
| 7 | M | 68 | ＋ | － | － | － | － |
| 8 | M | 68 | ＋ | － | － | － | － |
| 9 | M | 64 | ＋ | － | － | － | － |
| 10 | M | 64 | ＋ | － | － | － | － |
| 11 | F | 47 | － | － | － | － | － |
| 12 | M | 51 | － | － | － | － | － |
| 13 | F | 60 | ＋ | － | － | － | － |
| 14 | F | 63 | ＋ | － | － | － | － |
| 15 | F | 66 | ＋ | － | － | － | － |
| 16 | M | 65 | ＋ | － | － | － | － |
| 17 | M | 71 | ＋ | － | － | － | － |
| 18 | F | 76 | ＋ | － | － | － | － |
| 19 | M | 69 | ＋ | ＋ | － | － | － |
| 20 | M | 58 | ＋ | － | － | － | － |
| 21 | M | 54 | － | － | － | － | － |
| 22 | F | 55 | － | － | ＋ | ＋ | ＋ |
| 23 | F | 55 | ＋ | － | － | － | － |
| 24 | M | 54 | ＋ | － | － | － | － |
| 25 | M | 62 | ＋ | － | － | － | － |
| 26 | M | 77 | ＋ | － | ＋ | ＋ | ＋ |
| 27 | M | 63 | ＋ | － | － | － | － |
| 28 | M | 56 | － | － | － | － | － |
| 29 | F | 59 | ＋ | － | － | － | － |

| Case No. | FFA | | | | | ICGA | | | | | |
| --- | --- | --- | --- | --- | --- | --- | --- | --- | --- | --- | --- |
|  | Occult CNV | Transmitted  fluorescence | PED | NRD | CME | Polypoidal lesions | Branching  vascular networks | PED | NRD | CME | late phase  hypofluorescence region |
| 1 | ＋ | － | ＋ | － | － | ＋ | ＋ | － | － | － | － |
| 2 | ＋ | － | ＋ | － | － | ＋ | ＋ | － | － | － | － |
| 3 | ＋ | － | － | － | － | ＋ | － | － | － | － | － |
| 4 | ＋ | ＋ | ＋ | － | － | ＋ | ＋ | ＋ | － | － | ＋ |
| 5 | ＋ | － | － | － | － | ＋ | － | － | － | － | － |
| 6 | ＋ | － | ＋ | － | － | ＋ | ＋ | ＋ | － | － | － |
| 7 | ＋ | ＋ | ＋ | － | － | ＋ | － | － | － | － | - |
| 8 | ＋ | － | ＋ | － | ＋ | ＋ | ＋ | ＋ | － | － | － |
| 9 | ＋ | － | － | － | － | ＋ | － | － | － | － | － |
| 10 | ＋ | － | ＋ | － | － | ＋ | ＋ | ＋ | － | － | － |
| 11 | ＋ | － | － | － | － | ＋ | ＋ | ＋ | － | － | － |
| 12 | ＋ | － | ＋ | － | － | ＋ | － | － | － | － | － |
| 13 | ＋ | － | ＋ | ＋ | ＋ | ＋ | ＋ | ＋ | ＋ | － | － |
| 14 | ＋ | － | ＋ | － | － | ＋ | － | ＋ | － | － | － |
| 15 | ＋ | ＋ | － | － | － | ＋ | － | － | － | － | ＋ |
| 16 | ＋ | － | － | － | － | ＋ | ＋ | － | － | － | － |
| 17 | ＋ | ＋ | ＋ | － | － | ＋ | ＋ | ＋ | － | － | - |
| 18 | ＋ | － | － | － | － | ＋ | － | － | － | － | － |
| 19 | ＋ | － | ＋ | － | － | ＋ | ＋ | － | － | － | － |
| 20 | ＋ | ＋ | － | － | － | ＋ | － | － | － | － | - |
| 21 | ＋ | － | － | － | － | ＋ | ＋ | ＋ | － | － | － |
| 22 | ＋ | － | － | － | － | ＋ | ＋ | － | － | － | － |
| 23 | ＋ | － | － | － | － | ＋ | － | － | － | － | － |
| 24 | ＋ | － | ＋ | － | － | ＋ | ＋ | ＋ | － | － | － |
| 25 | ＋ | ＋ | ＋ | ＋ | ＋ | ＋ | ＋ | － | － | － | - |
| 26 | ＋ | ＋ | ＋ | － | － | ＋ | － | ＋ | － | － | ＋ |
| 27 | ＋ | － | － | － | － | ＋ | － | － | － | － | － |
| 28 | ＋ | － | － | － | － | ＋ | ＋ | ＋ | － | － | － |
| 29 | ＋ | ＋ | ＋ | － | － | ＋ | ＋ | － | － | － | - |

| Case No. | SD-OCT | | | | | Retro mode | | | | | | |
| --- | --- | --- | --- | --- | --- | --- | --- | --- | --- | --- | --- | --- |
|  | PED | NRD | CME | Drusen | minute RPE  protrusion | Polypoidal  lesions | Branching  vascular networks | PED | NRD | CME | Drusen | minute granular  RPE changes |
| 1 | ＋ | ＋ | － | － | － | ＋ | ＋ | ＋ | － | － | － | － |
| 2 | ＋ | － | － | － | － | ＋ | ＋ | ＋ | － | － | － | － |
| 3 | － | － | － | － | － | ＋ | － | － | － | － | － | － |
| 4 | ＋ | － | － | － | ＋ | ＋ | ＋ | ＋ | － | － | － | ＋ |
| 5 | ＋ | － | － | － | － | ＋ | － | － | － | － | － | ＋ |
| 6 | ＋ | － | － | － | － | ＋ | ＋ | ＋ | － | － | － | － |
| 7 | ＋ | － | － | － | ＋ | ＋ | － | ＋ | － | － | － | ＋ |
| 8 | ＋ | ＋ | ＋ | － | － | ＋ | ＋ | ＋ | ＋ | ＋ | － | － |
| 9 | － | － | － | ＋ | ＋ | ＋ | － | － | － | － | ＋ | ＋ |
| 10 | ＋ | － | － | － | － | ＋ | ＋ | ＋ | － | － | － | － |
| 11 | ＋ | － | － | － | － | ＋ | ＋ | ＋ | － | － | － | － |
| 12 | ＋ | － | － | ＋ | ＋ | ＋ | － | ＋ | － | － | ＋ | ＋ |
| 13 | ＋ | ＋ | ＋ | － | － | － | ＋ | ＋ | ＋ | ＋ | － | － |
| 14 | ＋ | － | － | － | － | ＋ | － | ＋ | － | － | － | － |
| 15 | － | － | － | － | ＋ | ＋ | － | － | － | － | － | ＋ |
| 16 | ＋ | － | － | － | － | ＋ | ＋ | ＋ | － | － | － | － |
| 17 | ＋ | － | － | － | ＋ | ＋ | ＋ | ＋ | － | － | － | ＋ |
| 18 | － | － | － | － | － | ＋ | － | － | － | － | － | － |
| 19 | ＋ | － | － | － | － | ＋ | ＋ | ＋ | － | － | － | － |
| 20 | － | － | － | ＋ | ＋ | ＋ | － | － | － | － | ＋ | ＋ |
| 21 | ＋ | － | － | － | － | ＋ | ＋ | ＋ | － | － | － | － |
| 22 | ＋ | － | － | － | － | ＋ | ＋ | － | － | － | － | ＋ |
| 23 | － | － | － | － | － | ＋ | － | － | － | － | － | － |
| 24 | ＋ | ＋ | － | － | － | － | － | ＋ | － | － | － | － |
| 25 | ＋ | ＋ | ＋ | ＋ | ＋ | ＋ | ＋ | ＋ | ＋ | ＋ | ＋ | ＋ |
| 26 | ＋ | － | － | － | ＋ | ＋ | － | ＋ | － | － | － | ＋ |
| 27 | － | － | － | － | － | ＋ | － | － | － | － | － | － |
| 28 | ＋ | － | － | － | － | ＋ | ＋ | ＋ | － | － | － | － |
| 29 | ＋ | － | － | － | ＋ | ＋ | ＋ | ＋ | － | － | － | ＋ |

FAF=fundus autofluorescence; FFA=fundus fluorescein angiography; ICGA=indocyanine green angiography; SD-OCT=Spectral-domain optical coherence tomography; CNV=choroidal neovascularization; PED=pigment epithelial detachment; NRD=neuroretinal detachment; CME=cystoid macular edema; RPE=retinal pigment epithelium; M=man; F=female
